# Rheumatoid Vasculitis Complicated With Acute Inflammatory Demyelinating Polyneuropathy in an Older Female: A Case Report

**DOI:** 10.7759/cureus.38214

**Published:** 2023-04-27

**Authors:** Ryuichi Ohta, Hirotaka Ikeda, Chiaki Sano

**Affiliations:** 1 Community Care, Unnan City Hospital, Unnan, JPN; 2 Community Medicine Management, Shimane University Faculty of Medicine, Izumo, JPN

**Keywords:** japan, general medicine, rural hospital, guillain–barré syndrome, rheumatoid vasculitis

## Abstract

Rheumatoid vasculitis (RV) causes various complications in the heart, lungs, kidneys, and nerves that require intensive treatment. Rapid RV-related peripheral nerve involvement progression is critical and requires prompt treatment. We report the case of a 73-year-old female with RV, with a chief complaint of difficulty walking without any infectious symptoms for several months. We diagnosed Guillain-Barré syndrome (GBS) accompanied by RV and treated the patient with intravenous immunoglobulin and cyclophosphamide. Previous impairments of activities of daily living (ADLs) were resolved. Diagnosing the neurological manifestations of RV and GBS in older patients with an active RV is challenging because of the various patterns of the progression. For effective management, considering both diseases and implementing immunosuppressive and modulatory treatments is critical to stop the progression of neurological symptoms and prevent the deterioration of ADLs.

## Introduction

Rheumatoid vasculitis (RV), a form of advanced rheumatoid arthritis, causes systemic vasculitis with various complications involving the heart, lungs, kidneys, and nerves and requires intensive immunosuppressive treatments [1]. Neurological involvement in RV shows acute and chronic progressive symptoms in the central and peripheral nervous systems [2]. The neurological symptoms of RV are mainly treated with intravenous immunoglobulin (IVIG) and cyclophosphamide (CYC) [3]. Delayed treatment of RV can affect the activities of daily living (ADLs), such as walking and toileting. Guillain-Barré syndrome (GBS) is an acute progressive peripheral neuropathy [4], mainly caused by post-infectious autoimmune processes [5]. From the clinical courses, the differentiation between rapid RV-related peripheral nerve involvement progression and GBS is challenging. However, there are few reports regarding the coexistence of GBS and RV [6]. We treated an older female patient with RV with a chief complaint of difficulty walking without any infectious symptoms for several months. Eventually, the patient was diagnosed with GBS, treated with IVIG and CYC, and cured of ADLs. This case report discusses the possible relationship between RV progression and GBS.

## Case presentation

A 73-year-old female presented to a rural community hospital with a chief complaint of difficulty walking for two weeks. Four years prior, she had tested positive for rheumatoid factor and anti-citrullinated protein antibodies and was diagnosed with rheumatoid arthritis with polyarthritis of the hands. The patient was treated with methotrexate (12 mg/week) to achieve clinical remission. Three months before admission, her joint pain had exacerbated in both hands, and her deformities had progressed. After ruling out bacteremia and septic arthritis, prednisolone (5 mg/day) was administered. One month before admission, she experienced progressive dyspnea and was diagnosed with pericarditis and acute heart failure without new valvular changes. Considering progressive rheumatoid arthritis, pericarditis, and heart failure, she was diagnosed with possible RV and treated with prednisolone (30 mg/day). Treatment alleviated joint and heart inflammation and restored ADLs. However, one week before admission, weakness in both legs gradually began and progressed without any predisposing factors, deteriorating her ADLs, such as standing up and moving to chairs and toilet by herself. She visited a rural community hospital because of difficulty walking. She had no infection symptoms in a few months without back and leg pain. Past medical history included right ventricular hypertension without diabetes and any vitamin deficiencies. Her annual health checkup did not show any malignancy. The medications administered included enalapril 5 mg daily, methotrexate 12 mg/week, and prednisolone 30 mg daily.

The vital signs at the visit were as follows: blood pressure, 113/57 mmHg; pulse rate, 86 beats/minute; body temperature, 36.7°C; respiratory rate, 14 breaths/minute; and oxygen saturation, 98% on room air. The patient was alert to time, place, and person. Physical examination revealed that the bilateral knee and Achilles reflexes had disappeared, and the lower extremities were weak. Manual muscle testing findings were as follows: bilateral upper extremities, 5/5 (right/left); iliopsoas, 4/4 (right/left); quadriceps, 4/4 (right/left); triceps, 3/4 (right/left); gastrocnemius, 3/4 (right/left); tibialis anterior, 3/3 (right/left); extensor hallucis longus, 3/3 (right/left); and flexor hallucis longus, 3/3 (right/left). No obvious abnormalities were observed on the chest, abdomen, or skin. Laboratory tests revealed elevated inflammatory marker levels, anemia, and leukocytopenia (Table 1).

**Table 1:** Initial laboratory data of the patient. eGFR: estimated glomerular filtration rate; CRP: C-reactive protein; Ig: immunoglobulin; SARS-CoV-2: severe acute respiratory syndrome coronavirus 2; C3: complement component 3; C4: complement component 4; MPO-ANCA: myeloperoxidase anti-neutrophil cytoplasmic antibody; SS: Sjögren’s syndrome

Parameter	Level	Reference
White blood cells	8.00	3.5–9.1 × 10^3^/μL
Neutrophils	82.9	44.0–72.0%
Lymphocytes	6.7	18.0–59.0%
Monocytes	10.0	0.0–12.0%
Eosinophils	0.1	0.0–10.0%
Basophils	0.3	0.0–3.0%
Red blood cells	2.85	3.76–5.50 × 10^6^/μL
Hemoglobin	10.0	11.3–15.2 g/dL
Hematocrit	30.0	33.4–44.9%
Mean corpuscular volume	105.0	79.0–100.0 fL
Platelets	17.0	13.0–36.9 × 10^4^/μL
Erythrocyte sedimentation rate	34	2–10 mm/hour
Total protein	7.5	6.5–8.3 g/dL
Albumin	3.3	3.8–5.3 g/dL
Total bilirubin	1.2	0.2–1.2 mg/dL
Aspartate aminotransferase	19	8–38 IU/L
Alanine aminotransferase	19	4–43 IU/L
Alkaline phosphatase	137	106–322 U/L
γ-Glutamyl transpeptidase	67	<48 IU/L
Lactate dehydrogenase	513	121–245 U/L
Blood urea nitrogen	44.5	8–20 mg/dL
Creatinine	0.85	0.40–1.10 mg/dL
eGFR	50.1	>60.0 mL/minute/L
Serum Na	140	135–150 mEq/L
Serum K	3.1	3.5–5.3 mEq/L
Serum Cl	101	98–110 mEq/L
Serum Ca	9.1	3.5–5.3 mg/dL
Serum P	3.7	0.2–1.2 mg/dL
Serum Mg	2.0	1.8–2.3 mg/dL
CRP	10.35	<0.30 mg/dL
IgG	1583	870–1,700 mg/dL
IgM	575	35–220 mg/dL
IgA	55	110–410 mg/dL
IgE	30	<173 mg/dL
Syphilis Treponema antibody	0.00	S/CO
SARS-CoV-2 antigen	-	
Anti-nuclear antibody	40	<40
C3	125	86–164 mg/dL
C4	27	17–45 mg/dL
MPO-ANCA	<1.0	<3.5 U/mL
anti-SS-A/Ro antibody	<1.0	<10.0 U/mL
Urine test		
Leukocyte	(1+)	
Nitrite	(-)	
Protein	(1+)	
Glucose	(-)	
Urobilinogen	normal	
Bilirubin	(-)	
Ketone	(-)	
Blood	(+-)	
pH	6.5	
Specific gravity	1.013	
Cerebrospinal fluid		
Cell	0	0–5/μL
Protein	59	15–45 mg/dL
Glucose	56	48–83 mg/dL

**Figure 1:**
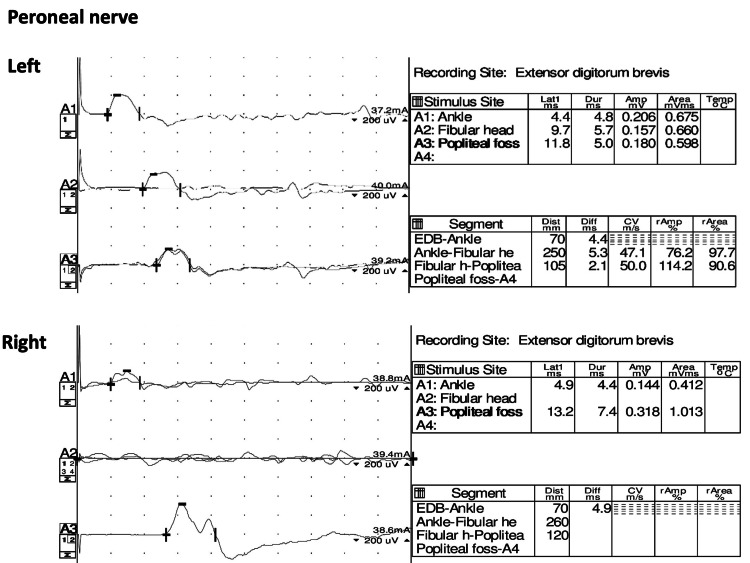
Peroneal nerve conduction velocity tests showing the possibility of demyelination and axonal injury.

Brain and lumbar magnetic resonance imaging revealed no evidence of stroke or inflammation. A cerebrospinal fluid (CSF) test revealed elevated CSF protein levels, suggesting acute demyelination (Table 1). Considering the possibility of GBS, antiganglioside antibody tests (GM1 and GQ1b antibodies) were performed. Peroneal nerve conduction velocity tests revealed possible demyelination and axonal injury (Figure 1).

**Figure 2:**
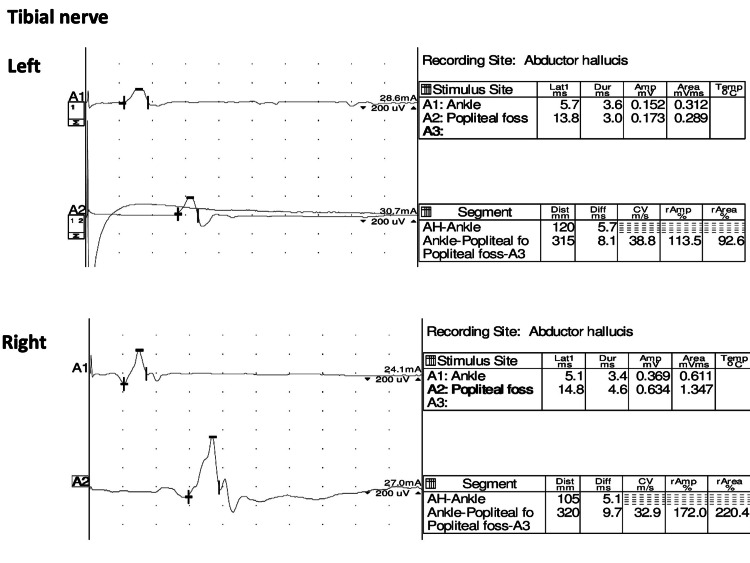
Nerve conduction velocity tests of tibial nerves showing the possibility of demyelination and axonal injury.

Nerve conduction velocity tests of the tibial nerves revealed possible demyelination and axonal injury (Figure 2).

Based on the clinical findings, the patient was diagnosed with possible GBS and peripheral neuropathic manifestations of the RV. The patient was administered IVIG 16 g/day for five days and prednisolone 1 g/day for three days. Prednisolone was tapered to 20 mg/day, and intravenous CYC 500 mg was used for both GBS and RV exacerbation. Seven days later, the patient’s symptoms and inflammatory condition were alleviated. Considering the rapid alleviation of the neurological symptoms, a diagnosis of neurological manifestations of RV was considered. However, on day 10 of admission, positive anti-GM1 antibody test results (>3.00) and negative anti-GQ1b test results suggested GBS. Two weeks after CYC treatment, azathioprine 25 mg/day was initiated and titrated to 50 mg/day within two weeks. The prednisolone dose was tapered to 10 mg/day for the same duration. Her ADLs were normal, and she was discharged home. At the three-month outpatient follow-up, her ADLs continued to be normal, and during the three months, the prednisolone dose was tapered to 3 mg and azathioprine 50 mg daily.

## Discussion

This case report presents the challenges in diagnosing the neurological manifestations of RV and GBS in an older patient with active RV. For effective management, considering both diseases and implementing immunosuppressive and modulatory treatments is critical to stop the progression of neurological symptoms and prevent the deterioration of ADLs.

Differentiating between the neurological manifestations of RV and GBS is challenging when the neurological symptoms appear shortly after RV diagnosis. In this case, the preceding diagnosis of RV made it difficult to differentiate between the neurological manifestations of RV and GBS. GBS is generally caused by preceding infections or medication usage [5,7]. However, the patient did not show any symptoms of infection or use of new medications. In addition, RV progressed during the clinical course, even with a moderate prednisolone dose [8]. The progression of both diseases can interfere with patients’ ADLs, and prompt investigation of nerve conduction velocity tests cannot distinguish between them; therefore, empirical treatments for both conditions are inevitable [9,10].

Considering the possibility of RV exacerbation and the development of GBS, implementing immunosuppressive and modulating treatments is essential to prevent the progression of both conditions in older patients with RV. In our case, differentiating between exacerbated RV and GBS development was challenging because of the atypical development of GBS. Eventually, prompt treatment, including both diseases, benefited patients regaining previous ADLs. It can be important not to rely on typical infectious symptoms to diagnose GBS among immunosuppressive patients. However, treating both conditions is invasive and requires shared decision-making [11]. Older patients with advanced rheumatoid arthritis can develop RV and require intensive treatment; therefore, prompt investigation of RV and its complications is essential [1,2,6]. In addition, as this case shows, neurological complications of RV can be similar to GBS in atypical presentations as they both can rapidly progress to rapid ascending neuropathy. Rheumatologists should be aware of both pathophysiologies, consider the possibility of GBS development, and identify order-specific antibodies associated with GBS. In addition, the diagnosis should be established through the follow-up of the effect of treatments for protecting older people’s ADLs.

Clinical progression and nerve conduction velocity test results can be beneficial for considering the coexistence of RV and GBS. Regarding the clinical progression, GBS can show an acute progressive course, typically manifesting as bilateral lower leg paralysis with acute deterioration [6]. RV may show acute and chronic progressive course, typically unilateral lower leg paralysis but possibly bilateral presentations with acute deterioration [4,12]. In our case, regarding the speed of progression, the presentation can fit both RV exacerbation and GBS diagnosis. In contrast, nerve conduction velocity tests revealed demyelination and axonal injury, possibly with acute and chronic progressive patterns. As observed in this case, a chronic progressive pattern may increase RV possibility. The findings of clinical progression and results of nerve conduction velocity tests can benefit patients with acute leg paralysis caused by vascular and inflammatory diseases and show the coexistence of RV and GBS, especially when patients do not have preceding infections.

## Conclusions

Diagnosing the neurological manifestations of RV and GBS in older patients with an active RV is challenging. For effective management, considering both diseases and implementing immunosuppressive and modulatory treatments are critical for stopping the progression of neurological symptoms and preventing the deterioration of ADLs.
